# Mendel-200: Pea as a model system to analyze hormone-mediated stem elongation

**DOI:** 10.1080/15592324.2023.2207845

**Published:** 2023-05-11

**Authors:** Ulrich Kutschera, Rajnish Khanna

**Affiliations:** I- Cultiver, Inc, Manteca, CA 95336 & Department of Plant Biology, Carnegie Institution for Science, Stanford, CA, USA

**Keywords:** Cell elongation, garden pea, gibberellin action, *Pisum sativum*, sucrose metabolism

## Abstract

In a recent Review Article on Gregor Mendel’s (1822–1884) work with pea (*Pisum sativum*)-plants, it was proposed that this crop species should be re-vitalized as a model organism for the study of cell- and organ growth. Here, we describe the effect of exogenous gibberellic acid (GA_3_) on the growth of the second internode in 4-day-old light-grown pea seedlings (*Pisum sativum*, large var. “Senator”). lnjection of glucose into the internode caused a growth-promoting effect similar to that of the hormone GA_3_. Imbibition of dry pea seeds in GA_3_, or water as control, resulted in a drastic enhancement in organ development in this tall variety. Similar results were reported for dwarf peas. These “classical” experimental protocols are suitable to study the elusive effect of gibberellins (which act in coordination with auxin) on the regulation of plant development at the biochemical and molecular levels.

In 1866, the Austrian abbot and biologist Gregor Mendel (1822–1884) published a Research paper^1^ entitled “Versuche über Pflanzenhybriden (Experiments on Plant Hybrids)”. With reference to earlier work of Joseph Kölreuter (1733–1806) and other botanists, who had discovered “Father-mother (male-female)-type sexuality in plants^2^, Mendel used populations of the Garden Pea (*Pisum sativum* L.) as model organism in his attempts to elucidate the principles of inheritance (Figure 1).

**Figure 1.**
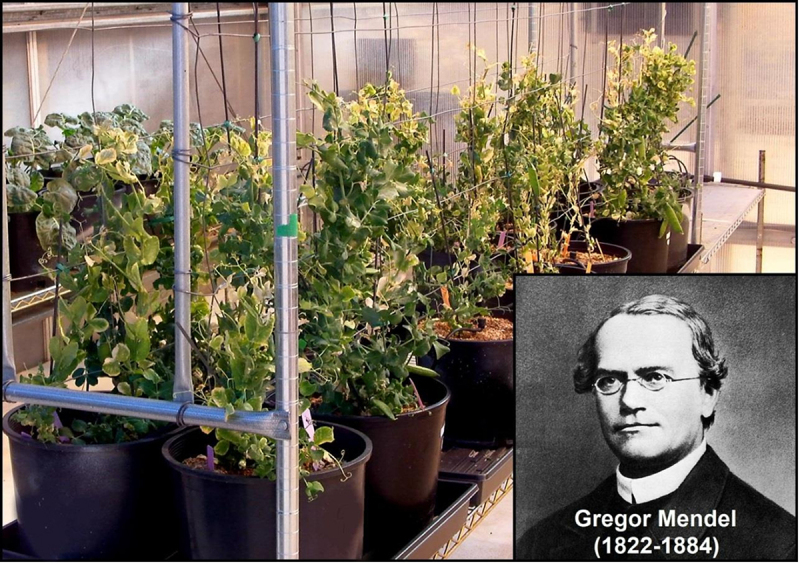
Portrait of the Austrian scientist Gregor Mendel and adult Garden Pea (*Pisum sativum*)-plants, cultivated in California (Original photo U. Kutschera, Carnegie Institution for Science, Stanford, CA 94305-USA, 2018).

By focusing on seven clearly distinguishable traits (1. Stem length: long vs. short; 2. flower color: purple vs. white; 3. Flower position: axial vs. terminal; 4. seed shape: round vs. wrinkled; 5. seed color: yellow vs. green; 6. Pod shape: inflated vs. constricted; 7. Pod color: yellow vs. green), he was able to deduce the concept of dominant-recessive inheritance of characteristics, and to formulate what we today call “Mendel’s Laws”.

In a *Review Article* entitled “Mendel: From genes to genome”, Sussmilch et al.^3^ documented that, in 2022, four of Mendel’s seven genes had been characterized, and that candidates of the three remaining genes have been proposed. As pointed out by these authors, the stem length Tall-vs.-dwarf *(LE* vs. *le*)-gene locus has been used in agriculture over hundreds of years. In this context, the short-internode dwarf (*le*) pea varieties, characterized by retarded stem growth, displayed a reduced tendency for lodging, and susceptibility for disease. Hence, short pea plants (for instance, “Meteor”) were of superior suitability for agricultural purposes. In 1997, it was shown that the *LE*-gene encodes a Gibberellin-3-oxidase (PsGA3ox1)^4^ that functions as an activator of the inactive precursor GA_20_, resulting in the active plant hormone Gibberellin 1 (GA_1_). This step is regulated by auxin (IAA, Indole-3-acetic acid), so that GA_1_ mediates growth-stimulation via IAA, which promotes the biosynthesis of active gibberellins (Figure 2). Based on these and other insights, Sussmilch et al.^3^ proposed to “re-vitalize pea as an excellent model species for physiological-genetic-studies”.

**Figure 2.**
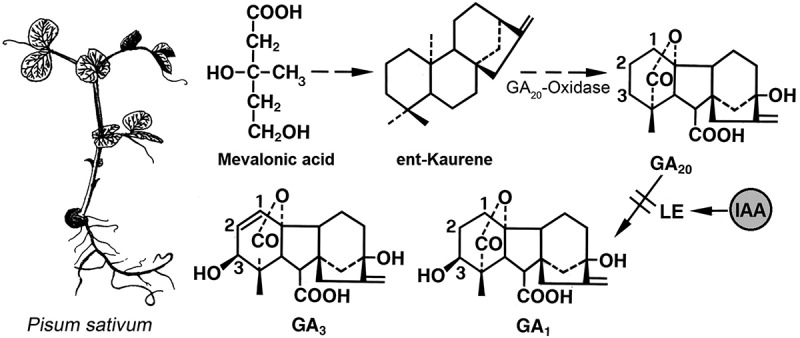
Developing pea-seedling (left) and simplified scheme of the biosynthetic pathway leading to the active Gibberellin GA_1_ (right). Chemical structure of the commercially available substance Gibberellic acid (GA_3_) is shown. LE = mutation leading to the occurrence of active gibberellin (GA_1_). IAA = Indole-3-acetic acid, i.e. auxin, which can promote GA_1_-biosynthesis (adapted from ref. 4).

In this *Commentary*, we present protocols and data showing that populations of seedlings of the Garden Pea may be employed as a system for the experimental analysis of the mechanisms of stem elongation.

It has long been known that, in gibberellin-deficient mutants of *P. sativum*, exogenously applied gibberellic acid (GA_3_) can restore the rapid stem growth observed in tall, wild-type plants^5,6^. However, the exact mechanism(s) by which this phytohormone regulates organ development is still elusive.

Five decades ago, Broughton and McComb^7^ reported that injection of glucose into pea stems simulates the growth-promoting action of GA_3_. The authors used the dwarf pea cultivar “Meteor”. To our knowledge, this important experiment has never been repeated with a tall variety of the garden pea. Therefore, we carried out the investigations summarized in Figure 3 A, using the protocols described in ref. 7. Four-day-old pea seedlings (large var. “Senator”) of uniform size were selected. Treatment consisted of injection of 5 µl sterile glucose (0,56 mol/L) or GA_3_ (0,75 mmol/L)-solution into the second internode, using a Microliter-Syringe. In the corresponding controls, distilled water (5 µL) was injected. The seedlings were grown in a controlled-environment chamber (23 to 27°C; 80% relative humidity) with a 12 h : 12 h (light : dark) schedule and a photosynthetic photon flux density of 150 µmol m^−2^ s^−1^ at plant level.

**Figure 3.**
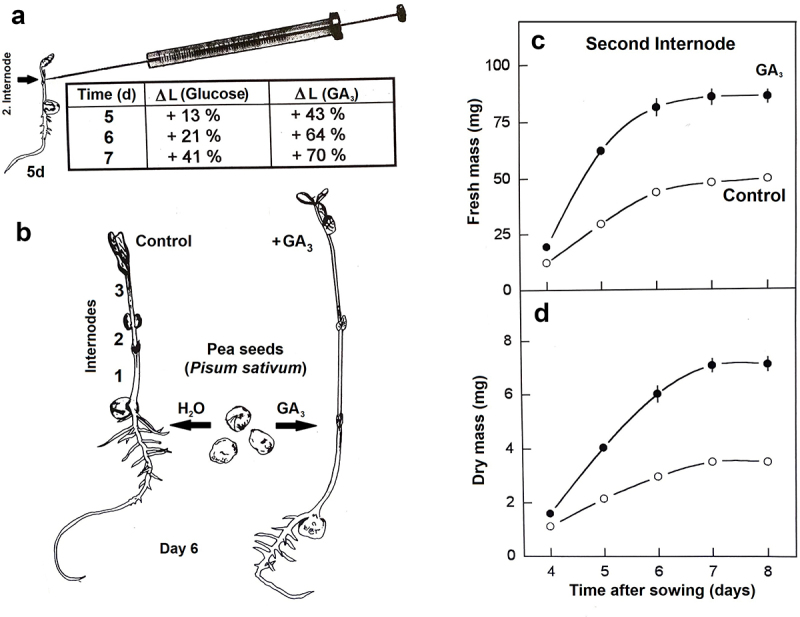
Experimental analysis of the mode of action of Gibberellic acid (GA_3_) in pea seedlings (*P. sativum*, large var. “Senator”). Glucose- or GA_3_-injection into the 2. internode, and representative data on stem growth (A). Protocol for the study of the effect of GA_3_ on plant development via imbibition of dry seeds (B). Effect of Gibberellic Acid (GA_3_) on changes in fresh- and dry mass of the second internode. Data points are based on six independent experiments each (C, D) (adapted from ref. 5 and unpublished results).

On day 5, the lengths of the second internodes were measured (± glucose or GA_3_, respectively) and the increase calculated (%). This treatment was repeated on days 5 and 6. Injection of sterile glucose into the second internode of young “Senator” peas caused significant elongation compared with the water control. However, GA_3_ had a much larger effect on the growth of this organ. This result demonstrates that, in tall “Senator” peas, glucose can only mimic part of the GA-effect on stem elongation. More work is required to resolve the question whether or not other pea-varieties respond like “var. Meteor”, or like the “Senator”-seedlings used here (Figure 3 A).

In the next set of experiments, we reexamined the effect of GA_3_, provided during the first hours of contact of dry seeds with water, on stem growth in a large variety. Seeds of *P. sativum* L. cv. “Senator” were imbibed for 6 h in either distilled water (– GA_3_) or a solution of gibberellic acid (GA_3_, optimal conc. of 0.2 mmol/L) (+ GA_3_). The imbibed seeds were sown in wet vermiculate 3 cm apart at a depth of ca. 1 cm in open plastic trays, and raised as described^5^.

Harvests for determinations of growth, i.e., fresh- and dry mass-increase, were performed at the same time each day (between 8:15 and 8:45 AM). Internodes were counted with the cotyledons at node zero. Only the second internode of the developing pea epicotyl was investigated (Figure 3B).

A detailed analysis of internode development in pea epicotyls revealed that, during growth, both the length and diameter of the stem increased^8^. Therefore, the time course of growth of internode 2 was studied by the measurement of the fresh mass of excised organs at daily intervals. Figure 3 C shows that, between days 4 and 6 after sowing, a steady increase in fresh mass occurred. In GA_3_-treated seedlings the rate of organ growth was much larger than in the water control. The final size of internode 2 was achieved on day 6 after sowing. No further gain in fresh mass was observed in control- and GA_3_-treated internodes. Corresponding changes with time in the dry mass of internode 2 are shown in Figure 3 D. The rate of dry matter accumulation was approximately constant until day 6 after sowing, and thereafter declined. In seedlings that were treated with GA_3_, the rate of dry mass increase was more than twice that of the control. A comparison between the kinetics shown in Figs. 3 Cand Figs. 3 D reveals that, after cessation of growth (i.e. fresh mass increase), dry matter accumulation continued for one further day. This may reflect the thickening of the stem segment after cessation of elongation^8^. Hence, both dwarf (“Meteor”) and tall *P. sativum* (“Senator”) respond to GA-treatment with a drastic stimulation of growth.

Two mechanisms have been proposed to account for the growth-promoting effect of GA_3_ in pea stems. Lockhart^9^ concluded that the hormone increases the extensibility of the cell walls, and hence permits rapid turgor-driven cell elongation. More recent studies have confirmed and extended this idea^10^. A second hypothesis is based on experiments by Broughton and McComb^7^. These authors reported that injection of glucose into internodes of the dwarf pea variety “Meteor” can simulate the effect of GA_3_ on stem elongation. Since GA_3_ has a promotive effect on the level of the enzyme acid invertase, which cleaves sucrose into glucose and fructose, it was proposed that the hormone acts via an enhancement in the level of hexoses within the vacuoles of the expanding cells^7^. Despite the fact that this “solute-import hypothesis of GA- action” is supported by a number of more recent studies^5,6^, more work is required to elucidate the exact mode of action of GA_3_ in the regulation of stem development in *P. sativum* (Figure 3 A – D). As indicated by Hedden^4^ in a recent summary on gibberellin biosynthesis, the role of auxin (IAA), which promotes cell elongation^11,12^, should be elucidated with respect to GA-action. We suggest that our simple experimental system may be suitable to address this important open question, i.e. the interaction of IAA and GAs in the regulation of organ growth, and refer to the excellent *Review Articles* of Castro-Camba et al.^13,14^ on this topic.

In summary, we agree with Sussmilch et al.^3^ that, 200 years after Mendel’s death, the Garden Pea should be re-vitalized as model organism for more detailed analyses of plant development from a physiological-biochemical, as well a molecular, perspective. Both compact and large pea-varieties are suitable systems for such studies.

Finally, we want to point out that the “Gibberellin-*P. sativum*-system” was an integral part of the “Green Revolution” of the 1960s-70s that resulted in novel crop plants with enhanced yields and more sustainable food production^13,14^.
